# Analysis of the Secondary Phases Formed by Corrosion of U_3_Si_2_-Al Research Reactor Fuel Elements in the Presence of Chloride Rich Brines

**DOI:** 10.3390/ma11071121

**Published:** 2018-06-30

**Authors:** Andreas Neumann, Martina Klinkenberg, Hildegard Curtius

**Affiliations:** 1 Institute of Geoscience and Geography, Mineralogy & Geochemistry, Martin-Luther University Halle-Wittenberg, Von-Seckendorff-Platz 3, D-06120 Halle (Saale), Germany; 2 Institute of Energy and Climate Research, IEK-6 Nuclear Waste Management, Forschungszentrum Jülich GmbH, Wilhelm-Johnen-Strasse, D-52425 Jülich, Germany; m.klinkenberg@fz-juelich.de; 3 Building and Property Management, Forschungszentrum Jülich GmbH, Wilhelm-Johnen-Strasse, D-52425 Jülich, Germany; h.curtius@fz-juelich.de

**Keywords:** research reactor fuel element U_3_Si_2_-Al, spent nuclear fuel, corrosion, secondary phases, layered double hydroxides LDH, lesukite

## Abstract

Corrosion experiments with non-irradiated U_3_Si_2_-Al research reactor fuel samples were carried out in synthetic MgCl_2_-rich brine to identify and quantify the secondary phases because depending on their composition and on their amount, such compounds can act as a sink for the radionuclide release in final repositories. Within the experimental period of 100 days at 90 °C and anoxic conditions the U_3_Si_2_-Al fuel sample was completely disintegrated. The obtained solids were subdivided into different grain size fractions and non-ambient X-ray diffraction (XRD) was applied for their qualitative and quantitative phase analysis. The secondary phases consist of lesukite (aluminum chloro hydrate) and layered double hydroxides (LDH) with varying chemical compositions. Furthermore, iron, residues of non-corroded nuclear fuel (U_3_Si_2_), iron oxy hydroxides and chlorides were also observed. In addition to high amorphous contents (>45 wt %) hosting the uranium, the quantitative phase analysis showed, that LDH compounds and lesukite were the major crystalline phases. Scanning electron microscopy (SEM) and energy dispersive -Xray spectroscopy (EDS) confirmed the results of the XRD analysis. Elemental analysis revealed that U and Al were concentrated in the solids. However, most of the iron, added as Fe(II) aqueous species, remained in solution.

## 1. Introduction

Due to considerable long-term impacts on the environment and society the waste management of spent nuclear fuel (SNF) is one of the most challenging issues for which sustainable disposal solutions must be found [1,2,3]. Yet, SNF arises not only from nuclear power plants it is also accumulated in research reactors of which currently around 250 are globally in operation. The composition of the fuel elements of such facilities differs from the oxide fuel types (UO_2_) of power plants and often consists of uranium bearing metallic alloys which are dispersed in an aluminum matrix. However, radioactivity is produced likewise by fission of uranium and therefore minor actinides, plutonium, and fission products are also a critical feature of spent research reactor fuel elements. Nuclear waste management has thus to cover this type of high-level waste (HLW) on a scientific (e.g., [4,5,6,7,8,9,10,11,12,13,14,15]) and regulative basis (e.g., [16,17]) as well. In some cases, existing contracts with manufactures of research reactor fuel elements regulate the return shipment of medium enriched fuel types (research reactors FRG-1 in Geesthacht and BER II in Berlin).

However, spent fuel (highly enriched U_3_Si_2_-Al with U-235 > 90 wt %) from the research reactor FRM II in Munich is considered to be disposed of in a final repository for HLW waste. It is therefore necessary to store the irradiated U_3_Si_2_-Al fuel elements in massive iron bearing containers (CASTOR^®^ MTR2 casks or alternatively in modified BSK-3 spent fuel coquilles) [18,19].

The long-term performance of the waste package, which has hence to be evaluated, will not only be governed by its design, it also depends on the chemical and physical situation in the deep geologic repository. Considering the long term safety of approx. 10^6^ years, storage conditions may change over time. The safety assessment of final repositories has to consider processes, which will lead to an alteration of the disposed SNF. 

Among those, the formation of secondary phases is relevant and has to be investigated, because the corrosion products constitute a sink for the radionuclide release and define thus parameters for the source term. This study focuses on the identification and quantification of secondary phases which were retrieved by corrosion of non-irradiated U_3_Si_2_-Al research reactor fuel elements in MgCl_2_ rich brine (which accounts for a repository in salt formations). Such investigations are also important due to the fact that corrosion of aluminum dispersed fuels exhibit higher degradations rates than those being determined for UO_2_ fuels of commercial nuclear power plants.

Wiersma [9] investigated the corrosion of different (non- and irradiated) fuel types (UAl, UAlx, U_3_O_8_, and U_3_Si_2_ alloys and chemical compounds). The microstructural investigations assumed the formation of gibbsite, hydrargillite, or bayerite as corrosion products. Surface analysis revealed also the formation of boehmite (cf. also [15]).

Corrosion rates presented and reviewed by Hilton [20] refer, among other fuel types, to the interaction of UAl_x_- and U_x_Si_y_-based fuels only with pure water. Therein, it was reported that at 80 °C the corrosion of the Al component of this fuel type leads to the formation of boehmite (AlO(OH)). The reaction rates of UAl_x_-Al dispersion fuel in water were essentially the same as those of the aluminum alloys. The Arrhenius expression for the aluminum alloy-H_2_O reaction was determined as k*_linear_* = 4.29exp(32.8 ± 1.8 kJ/(molRT)) (where k has units of mg metal/cm^2^h) for temperatures ranging from 25 to 360 °C. At 80 °C the rate constant could be estimated as 0.11 g/(m^2^d). The reported behavior of U_x_Si_y_-Al based fuels differs considerably. Most of the defected fuel plates exhibited a reaction rate ~10^5^ times faster than the rate of aluminum alloys and 100–1000 times faster than uranium silicide inter-metallics. This observation may be due to freshly exposed metal (machined holes as defect source) and/or an accelerated crevice corrosion.

Kaminski et al. [12] determined a corrosion rate of 9.7 × 10^−2^ g/(m^2^d) for a UAl_x_-Al sample for repository relevant conditions; the experiments were carried out at 90 °C by dripping permanently EJ-13 modified well water (Yucca Mountain site) up to 183 d on the specimen. 

Although test conditions were different (accounting for different national disposal regulations/strategies) corrosion rates for research reactor fuel elements determined by Curtius et al. [21] are of similar magnitude. The experiments were carried out at 90 °C under anoxic conditions in the presence of Fe(II) with irradiated and non-irradiated samples in MgCl_2_ rich brine (U_3_Si_2_-Al_irr_: 4.24 × 10^−2^ g/(m^2^d), U_3_Si_2_-Al: 5.74 × 10^−2^ g/(m^2^d), UAl_x_-Al_irr_: 7.69 × 10^−3^ g/(m^2^d), UAl_x_-Al: 1.02 × 10^−3^ g/(m^2^d)) and in clay pore water (U_3_Si_2_-Al_irr_: 6.93 × 10^−2^ g/(m^2^d), U_3_Si_2_-Al: 2.36 × 10^−2^ g/(m^2^d), UAl_x_-Al_irr_: 1.05 × 10^−3^ g/(m^2^d), UAl_x_-Al: 2.68 × 10^−3^ g/(m^2^d)). This study showed that corrosion in MgCl_2_ rich brine is somewhat faster than it was observed in clay pore water. However, comparing irradiated U_3_Si_2_-Al/UAl_x_-Al fuels [21] with irradiated UO_2_ (2.36 × 10^−6^ g/(m^2^d) [22], the corrosion rate of the alloys was increased up to ~3–4 orders of magnitude. Both experiments considered corrosion in chloride rich solution, but the setup of Loida [22] differs in some aspects, considering fuel sample specifications, temperature (25 °C) and composition of the brine (NaCl_sat_) as well as the iron supply simulating the waste package. After an experimental period of 3.5 years the irradiated U_3_Si_2_-Al and UAl_x_-Al fuel samples were fully decomposed. This implies a very fast release of the radioactive inventory. Yet, the radio analytical investigations of the secondary phases of the U_3_Si_2_-Al_irr_/UAl_x_-Al_irr_ fuel sample corrosion showed that the long-lived ^234^U, ^238,239,240^Pu and ^241^Am isotopes were immobilized by the solids [21]. This is observed for MgCl_2_ rich brine and for Mont Terri clay pore water as well. 

For the phase specific evaluation, considering the retention capacity of the solids, complementary tests with non-irradiated fuel were performed to identify and quantify the secondary phases. Kaminski et al. [12,14] observed the formation of a silica-substituted hydrous aluminum gel layer on the sample surface. Additionally, dehydrated uranyl oxyhydroxides, schoepite ([(UO_2_)_4_|O|(OH)_6_]·6H_2_O), becquerelite (Ca(UO_2_)_6_O_4_(OH)6·8(H_2_O)) and colloids, prevailingly silica rich, were also formed. More than 99 wt % of the dissolved uranium was bound to the colloids which exhibit a diameter of some hundred nanometers. Mazeina et al. [23,24] carried out experiments with UAl_x_-Al fuels under reducing conditions (identically to Curtius et al. [21]) and observed the formation of the crystalline phases hydrotalcite, i.e., LDH (layered double hydroxides—common composition: [M^2+^_1−x_ M^3+^_x_(OH)_2_]^q+^(X^n−^)_q/n_·yH_2_O) and bischofite (MgCl_2_·6H_2_O). In a more recent study with UAl_x_-Al by Klinkenberg et al. [25] LDH was again observed, yet with varying chemical compositions. Additionally, lesukite, an aluminum hydroxy chloride hydrate, being described by Vergasova et al. [26] and Witzke [27], was also identified as a major phase. Small amounts of iron (III) oxy hydroxides and iron (II) chlorides were observed as well. Further accessories like metallic iron and residues of nuclear fuel were also present. The amorphous content mounts up to ~20 wt % for UAl_x_-Al. In these studies [25,28], it was shown that the stability of observed phases strongly depends on the post treatment, i.e., on the chosen liquid (water or isopropanol) for the retrieval of the secondary phases. After the corrosion experiment of the non-irradiated fuel sample was finished the residues which were treated with water showed different secondary phases compared to those being treated with isopropanol. This is especially valid for lesukite which was not observed as a secondary phase considering the water treatment. Instead, different aluminum hydroxides (boehmite, nordstrandite, gibbsite) were observed [21,28] and hence indicating that isopropanol is more beneficial for a post treatment of corrosion solids. 

Further studies [29] with UAl_x_-Al in the presence of standardized clay pore water (Mont Terri type [30]) were carried out. The secondary crystalline phases gypsum, bassanite, goethite, and boehmite were identified. Non-corroded leftovers of UAl_4_ were also observed. The amorphous content exceeded 80 wt % for the system UAl_x_-Al in clay pore solution.

In this study non-irradiated U_3_Si_2_-Al fuel elements were corroded in MgCl_2_ rich solution (salt host rock). Fe(II)_aq._ was also added to simulate the decomposition of the waste package. The experiments focus on the non-ambient laboratory XRD phase analysis of the secondary phases. Efforts were taken and unique experimental equipment was applied to prevent the secondary phases from alteration by oxidation during retrieving, treatment, and analysis.

## 2. Materials and Methods

### 2.1. Setup of the Corrosion Experiments and Sample Pre-Treatment

The corrosion experiment (static batch, 90 °C) with an U_3_Si_2_-Al sample was carried out under anoxic condition in standardized solution (brine 2, cf. [6]). The small cut non-irradiated fuel platelet (40.2 × 20.0 × 1.4 mm^3^, S/V = 15.8 cm^−1^) weighed totally 4.40 g (m(U) = 1.6 g, m(Al) = 2.30 g, m(Si) = 0.28 g). The U_3_Si_2_-Al fuel matrix was two-sided covered with an aluminum cladding. The sample was put into a glass autoclave with 400 mL of magnesium chloride rich brine. 10 g of FeCl_2_·H_2_O were added to the solution to simulate the corrosion of the iron bearing waste package. The vessel was tightly closed, put into a drying oven and was heated to 90 °C. The corrosion progress was monitored by a probe measuring the hydrogen pressure built up. pH was also recorded; at the beginning of the experiment pH was little more than 1 and reached after ~75 days a constant value between 4 and 5. More specific details concerning setup, data monitoring, and the fuel sample are described by Curtius et al. [21]. 

The hydrogen pressure built up in the autoclave due to reducing conditions was monitored to observe indirectly the corrosion progress. After 100 days no further increase of the pressure was observed and the secondary phases were retrieved out of the vessel. Efforts were taken that every work step considering sample retrieval, pre-treatment, and drying was carried out under argon atmosphere. Inert conditions were necessary to prevent the secondary phases from alteration by oxidation. The suspension has been retrieved and separated for the pre-treatment. One part was used for the grain size classification into the fractions >63 µm, 2–63 µm, and <2 µm. This was achieved by wet sieving with isopropanol to obtain the fraction >63 µm. The subdivision of the smaller fraction was carried out also in isopropanol by a sedimentation procedure according to Atterberg [31]. Additionally, an analogous treatment for retrieving the secondary phases carried out again according to the protocol as described by Klinkenberg et al. [25].

The remaining part of the retrieved suspension was used to determine the amount of U, Al, Ca, Si, and Fe. After centrifugation the supernatant was used for elemental analysis. To determine the U content a Liquid Scintillation Counter (LSC) TRI-CARB 2020 (PerkinElmer, Waltham, MA, USA) and an 𝛼-analyzer for α-spectrometry (Canberra-Packard GmbH, Schwadorf, Austria) were used. Al, Ca, Si, and Fe were analyzed by ICP-OES (inductively coupled plasma optical emission spectroscopy ELAN 6100 DRC (PerkinElmer, Waltham, MA, USA) with a TJA-IRIS instrument. A full description of these analytical procedures is given elsewhere [21,25]. The estimation of the water content of the untreated sample was determined by drying for one week at 105 °C in argon atmosphere in which 82.57% of the weight accounted for water.

### 2.2. X-ray Diffraction Analysis

The phase identification was evaluated with the DiffracPlus software from Bruker-AXS (Karlsruhe, Germany) by retrieving the powder diffraction file PDF-2 (ICDD Release 2007). The amount of each crystalline phase and the amorphous content was determined by the Rietveld method [32,33]. Therefore, an internal non-certified zincite (ZnO) standard (Merck, Darmstadt, Germany) of known weight has been added for the quantitative X-ray phase analysis (QPA). 

The applied structures, i.e., the retrieved CIF-Files of the ICSD (Inorganic Crystal Structure Database) of the identified phases are summarized in Table 1. Exceptionally for lesukite a structure did not exist. Therefore, a model was derived [28]. 

The diffractograms were recorded with a D8 diffractometer from Bruker-AXS (Karlsruhe, Germany). The space group and lattice parameters of lesukite and the secondary phase quantification have been computed with TOPAS [34,35] and BGMN [36]. Both programs use the fundamental parameter approach (FPA), i.e., the full diffractometer device function is thereby defined by the emission spectra of the X ray tube [37] and by the geometry of the beam path. The goniometer of the diffractometer features a θ–θ geometry. For the XRD measurements CuK_α_ radiation (λ_1_ = 1.54059 Å) at 40 kV and 40 mA was applied. Further details about the diffractometer setup are given elsewhere (cf. [28,37]).

For the analyses it was crucial to avoid a sample alteration due to oxidation during the measurements. Therefore, the samples were put into a climate chamber from MRI (Materials research instruments). This device has been purged permanently with nitrogen while the diffractograms were recorded at room temperature.

### 2.3. SEM/EDS Analysis

The morphology and the chemical composition of the secondary phases were investigated with a FEI Quanta 200 ESEM FEG (Hillsboro, OR, USA). The instrument was equipped with an Apollo X silicon drift detector (EDAX, Mahwah, NJ, USA) for energy-dispersive X-ray spectroscopic (EDS) measurements. The particles of the different grain size fraction were prepared on adhesive carbon tabs without any previous sputtering. The samples were analyzed in low vacuum mode (0.6 mbar) at 20 kV with spot size 4, and 10 mm working distance. The investigations were carried out with the large field low vacuum detector LFD for secondary electrons and BSED (backscattered electrons) detectors of the SEM device.

To account for micro-absorption [38] the particle dimensions of the different grain size fractions were also measured. Some micrograms of each fraction were thus suspended in isopropanol, sonicated for several minutes, and then prepared on non-adhesive carbon tabs. Based on the assumption of spherical particle shape, the average diameter was determined with the image analysis software EDAX Genesis V 6.2.

## 3. Results and Discussion

### 3.1. X-ray Analysis of the Secondary Phases

**Figure 1:**
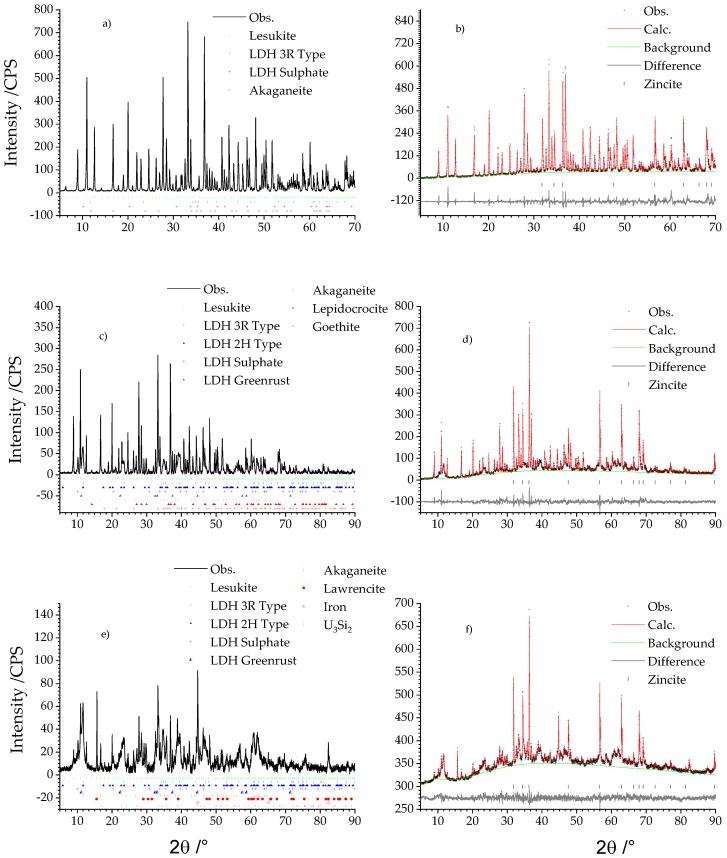
Qualitative (**left**) and quantitative (**right**) phase analyses of the secondary phases (**a**) Qualitative phase analysis of the fraction <2 µm, (**c**) qualitative phase analysis of the fraction 2–63 µm and (**e**) qualitative phase analysis of the fraction >63 µm. (**b**) QPA of the fraction <2 µm, (**d**) QPA of the fraction 2–63 µm, and (**f**) QPA of the fraction >63 µm.

Figure 1 shows the results of the qualitative and the quantitative phase analyses in dependence of the different grain size fraction <2 µm, 2–63 µm, and >63 µm. The qualitative phase analysis is given by the diffractograms shown left (Figure 1a,c,e). Results of quantitative phase analysis are given by the related Rietveld plots on the right column (Figure 1b,d,f).

The fraction <2 µm exhibited only three different compounds (cf. Table 1): akaganeite, lesukite, and two types of LDH of which one incorporated sulphate and the other chloride in the interlayer. This was inferred by analysing the respective (00l) basal reflections, which showed for sulphate intercalation an increase of the *d* spacing of adjacent layers normal to the *c* axis. The *d* spacing for the chloride type LDH is approx. 8 Å and approx. 8.6 Å for the sulphate type.

Lesukite was quantitatively the major phase and mounted up to 95.37 ± 0.31 wt %. Chloride and sulphate type LDH exhibit accessory amounts of 1.43 ± 0.26 and 0.25 ± 0.08 wt %. A further accessory mineral is akageneite with 2.05 ± 0.15 wt %. The amorphous content was practically not existent due to the determined uncertainties. 

The medium sized fraction from 2 to 63 µm featured additionally three different Fe(III) oxy hydroxides (akaganeite, goethite, lepidocrocite) and two new LDH compounds manasseite (2H LDH type) and greenrust (cf. Table 1). Quantitatively the grain size fraction 2–63 µm was still dominated by lesukite although its relative amount has nearly been halved to 51.00 ± 2.90 wt %. Approx. 30 wt % could be ascribed to the 3R (17.00 ± 4.30 wt %) and 2H (12.35 ± 1.40 wt %) LDH types. 3.94 ± 0.30 wt % was calculated for the sulphate LDH. The content of greenrust was very low (0.20 ± 0.06 wt %). Likewise, the Fe(III) oxy hydroxides: akaganeite (1.55 ± 0.16 wt %), goethite (0.53 ± 0.12 wt %), and lepidocrocite (0.73 ± 0.09 wt %). The amorphous content increased to 12.7 ± 5.30 wt %. 

In the fraction >63 µm (cf. Table 1) residues of non-corroded fuel with the composition U_3_Si_2_ could be observed. Lesukite and LDH compounds were still present. This was also valid for akaganeite. Moreover, Fe(0) and lawrencite (Fe(OH,Cl)_2_), were also observed. Compared to the fractions <63 µm the quantity of lesukite was again reduced and exhibited 13.87 ± 3.02 wt %. This trend is also valid for the LDH compounds: 3R-type (7.37 ± 1.58 wt %), 2H-type (10.21 ± 3.74 wt %), sulphate type (1.26 ± 0.45 wt %). However, the greenrust increased to 2.15 ± 0.51 wt %. Considering the iron oxy hydroxides, akaganeite was reduced to 0.65 ± 0.40 wt.%. Lepidocrocite and goethite were not present anymore. Iron (0.66 ± 0.23 wt %), lawrencite (0.68 ± 0.16 wt %), and U_3_Si_2_ were of equal but just of minor content. The amorphous part increased to 62.36 ± 2.74 wt % constituting the most abundant phase in the fraction >63 µm. This increase could be explained by the sample preparation. This fraction was obtained just by sieving whereas the smaller fraction was additionally subjected to the Atterberg procedure for the further grain size subdivision. Thereby, it could not be ruled out that amorphous parts which may have been present after sieving in the smaller fraction were dissolved during this application. This assumption is also valid for the UAlx-Al being subjected to MgCl_2_ rich solution [25] solution and clay pore water (Mont Terri type) [29]. 

Especially with respect to the study of UAl_x_-Al fuel in brine 2 [25,28] the system U_3_Si_2_-Al in brine 2 behaved similarly in many aspects. In both systems lesukite and LDH compounds are the major phases. Trace amounts of non-corroded fuel were also present and considerable amounts of amorphous phases were observed as well.

The occurrence of the different observed phases was also dependent on the grain size and showed a similar distribution (cf. [25,28]). Disregarding the observed residues of non-corroded nuclear fuel, other crystalline uranium bearing phases could neither be observed for the U_3_Si_2_-Al nor for the UAl_x_-Al system [25,28] in chloride rich solution. However, the disintegration of UAl_x_-Al fuel element in Mont Terri solution resulted in different corrosion behavior. Observed crystalline phases were goethite, calcium sulfates and residues of UAl_4_, yet most of the solids were amorphous compounds which represented the greatest solid part [29]. Therefore, composition and specific surface are critical parameters which will have an impact on the source term. The results of this study and the investigations of [21,25,28,29] generally support the assumption of a similar corrosion behavior of Al dispersed UAl_x_ and U_3_Si_2_ fuels (cf. [5]) and a faster corrosion of the aluminum component of Al based fuels was also affirmed because pure Al metal was not observed in the corrosion residues. 

**Figure 2:**
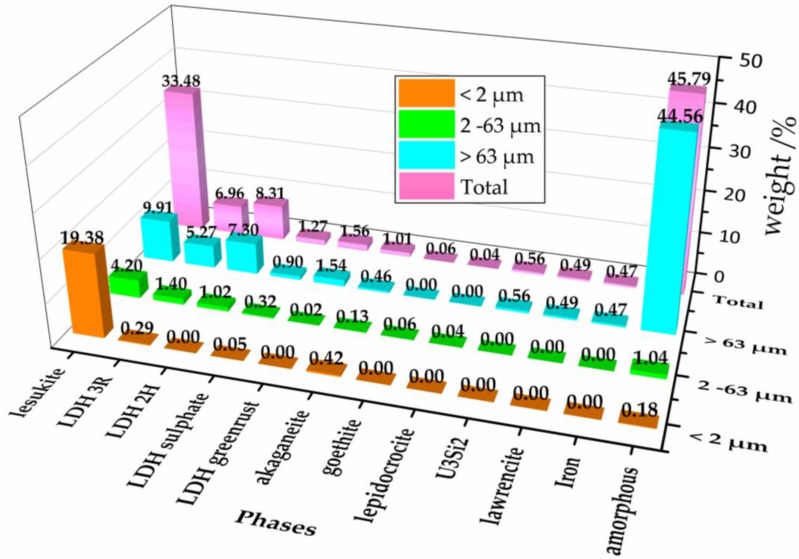
Quantitative phase distribution in dependence of the grain size fraction (Orange columns: <2 µm, green columns: 2–63 µm, light blue: >63 µm, and magenta: Total of all fractions).

Figure 2 shows an overview of the quantities being normalized for each fraction to the total amount of all obtained secondary phases. From the magenta colored columns in the last row, representing the total of all fractions could be seen that the amorphous part with more than 45 wt % is the most abundant phase. Second and third ranked in quantity were lesukite and various LDH types. All other phases were just present as accessories. It is expected that the amorphous part contains the uranium because—with exception of the residues of U_3_Si_2_—no further crystalline uranium phases were observed. The observed content of uranium and silicon (as U_3_Si_2_) was very low compared to the originally supplied quantities. With respect to the findings of [39] and taking into account that uranium was not found in the liquid part of the suspension of brine 2 it could be assumed that uranium is thus quantitatively constituents of the amorphous phases. This result constitutes an important finding as the analogue experiments of Curtius et al. [39] with irradiated U_3_Si_2_-Al showed that not only uranium but also americium, plutonium, and europium, as well were mostly immobilized in the solid phase. Therefore, possible implications for the source term must be evaluated whether the radionuclides in the SNF are also part of the amorphous phases, because the solubility is a critical parameter for their immobilization.

The ICP-OES results for aluminum and iron of the liquid phase of the corrosion products showed that iron is found in solution with 61.0 wt % (±0.5 wt %) whereas aluminum was totally part of the solid phase. Results indicated that the latter was part of crystalline and amorphous phase as well. Most of the magnesium was detected in the liquid phase due to the high solubility of MgCl_2_·6H_2_O which has been used for the preparation of the chloride rich brine. Yet, LDH phases contain considerable amounts of magnesium and constitute a major secondary phase. 

Contrary, silicon is mainly found the solid part. Most of silicon is probably part of the amorphous phase because the only crystalline Si bearing phases were remnants of U_3_Si_2_ which only host minor amounts of silicon by comparing its amount with original quantities of the non-corroded fuel sample. The elemental analysis of calcium indicated to be dissolved in the liquid phase. Neither it was observed in a crystalline secondary phase nor could calcium be detected in the solid phase. The proportion of sulphur (122.46 wt %) was slightly overestimated compared to its initially supplied amounts (100 wt %). Yet, this could be due to the uncertainties given by the very low originally supplied amounts (<0.007 g), by the sample quartering and preparation [21], and by the quantification (cf. Table 1). Nevertheless, from this finding it could be concluded that sulphur neither was a part of the amorphous phases nor has it been dissolved. It was totally fixed in the sulphate LDH. 

**Figure 3:**
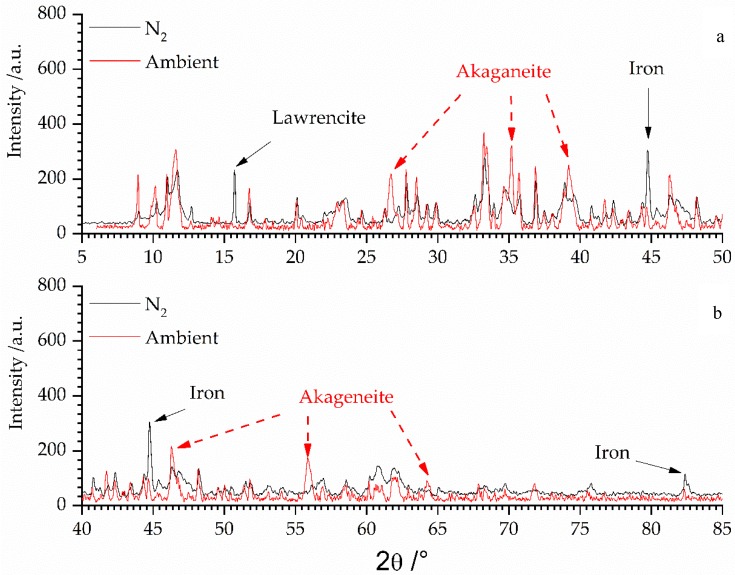
Comparison of diffractograms (divided for clarity’s sake in two ranges from 5–50° 2θ (**a**) and from 40–85° 2θ (**b**); overlap region between 40–50° 2θ) of fraction >63 µm being analyzed under inert conditions (N_2_—black line) and being subjected to ambient conditions (air: red line).

With respect to the safety assessment it is important to consider the phase stability of selected phases. The observed iron bearing phases exhibited valence states of 0, 2+, and 3+. One may interpret such a condition as a non-equilibrated system, yet artifacts due to preparation may also have an impact. Taking into account the quantitative development of the amorphous content of the different fractions it could not be ruled out that the Atterberg procedure gave reason for the observations made considering the iron valence state. Fe(0) and Fe(II) compounds (lawrencite, greenrust) were basically only observed in the fraction >63 µm whereas the fractions <63 µm are dominated by the Fe(III) compounds (akaganeite, goethite, and lepidocrocite). Although greenrust can accommodate Fe(II) as well, in the fraction 2–63 µm it was only of minor content. Therefore it could not be ruled out that during the Atterberg procedure Fe(0) and Fe(II) were oxidized although special care was taken to prevent the iron bearing secondary phases being altered. The impact of oxidation of Fe(0) and lawrencite (Fe(II)) of the fraction >63 µm is shown in Figure 3.

The black diffractogram, being recorded in N_2_ atmosphere, shows the fraction >63 µm (cf. Figure 1 and Figure 3). The peak positions of lawrencite and iron have been marked with black arrows. These reflections of the Fe^0/2+^ species vanished and new peaks (red arrows) of akaganeite (Fe^3+^) could be observed, when the sample was subjected to air for several weeks. 

### 3.2. SEM and EDS Analysis of the Secondary Phases

**Figure 4:**
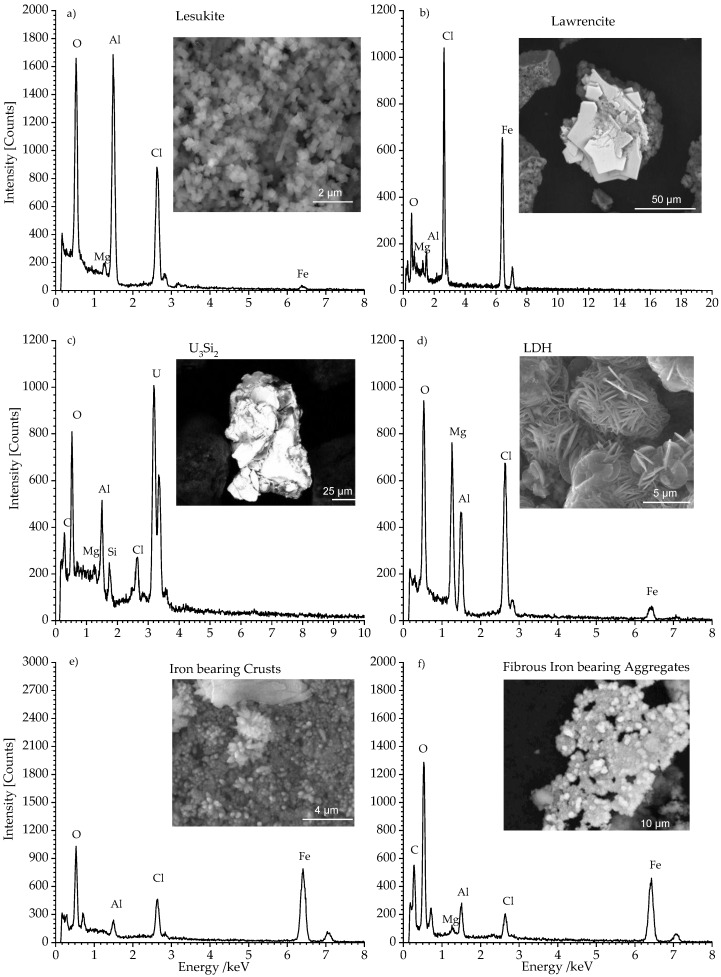
SEM (BSE) micrographs and EDS analyses of selected secondary phases: (**a**) shows cubic shaped lesukite, (**b**) shows platelets of Lawrencite FeCl_2_. (**c**) shows remnants of non-corroded U_3_Si_2_. (**d**) shows the typical sand rose like appearance of LDH phase. (**e**,**f**) show Iron bearing phases.

Figure 4a–f shows the SEM/EDS analyses of the pre-treated solid secondary phases obtained from the autoclave. In Figure 4a the observed compound exhibited perfectly cubic shaped lesukite crystals with an edge of several hundred nanometers. These crystals were observed in each grain size fractions. The related EDS spectrum showed typical lines of aluminum, chloride, and oxygen. This observation is in good agreement with the results reported by Vergasova et al. [26] and Witzke [27]. In Figure 4b lawrencite (FeCl_2_) is shown. This phase exhibited also platelet morphology. However, the crystals are ~10 times larger than the observed LDH compounds. The related EDS spectrum featured distinct iron and chlorine lines. This mineral belongs to the trigonal system and exhibited a layered structure which, contrary to LDH compounds (Figure 4d), did not feature any interlayer constituents (e.g., H_2_O, Cl^−^, SO_2_^4−^). The observed oxygen line could indicate quantitative exchange of chloride anions (r_shannon_ = 1.7 Å) by hydroxide anions (r_shannon_ = 1.4 Å). This assumption is based on the observation that the determined c lattice parameter—distance d of the layer spacing—is gradually smaller (5.62 Å) than the theoretical one (5.83 Å, cf. Table 1—ICSD code FeCl_2_: 64830 [40]). Figure 4c showed a very bright phase. This could be attributed to non-corroded leftovers of U_3_Si_2_-fuel. The respective EDS showed the expected signals for U, Si, and Al.

This phase was prevailingly located in the largest fraction >63 µm. Its presence was already evidenced in the related diffractogram (cf. Figure 1c). Traces of U_3_Si_2_ could also be observed in the fraction 2–63 µm.

Figure 4d shows sand rose shaped aggregations of laminar crystals. These platelet crystals had an in plane dimension of ~5 µm. Normal to these planes the thickness was clearly less than 0.5 µm. This phase was identified as a LDH-type compound. The EDS spectrum in Figure 4d exhibited the expected lines of magnesium, aluminum, chlorine, and oxygen. 

The LDH crystals are commonly observed in each grain size fraction (cf. Figure 1). The sulphate bearing LDH being clearly identified in the XRD analysis has not been observed by SEM/EDS analysis possibly due to the very low content of sulphate (cf. Table 1).

In Figure 4e the micrographs show iron compounds which formed coarse crusts. The crust formation was due to the sample desiccation especially in the fraction >63 µm. Fibrous aggregates were typically observed in fraction 2–63 µm (Figure 4f). Although Fe(III) oxy hydroxides (akaganeite, lepidocrocite, goethite), green rust, and Fe(0) have been observed in the diffractograms (cf. Figure 1), this phase did not show up in the related SEM/EDS analysis. This observation could be attributed to the poorly developed morphology and the small size of these iron bearing phases.

## 4. Conclusions

Within a short period (~100 days) the U_3_Si_2_-Al fuel sample corroded completely in the MgCl_2_ brine in the presence of Fe(II)_aq_. Elemental analysis (ICP-OES and LSC) showed that aluminum and uranium were quantitatively found in the secondary phases. Yet approx. 65 wt % of iron is remained in solution.

Special treatment was necessary for the characterization of the corrosion products, i.e., secondary phases were subdivided by sieving and by the Atterberg method in an inert atmosphere to prevent oxidative alteration, because XRD and SEM analyses revealed the presence of phases being sensitive to oxidation (iron, greenrust, and lawrencite, cf. Table 1).

As summarized in Table 1 the fraction <2 µm mainly consisted of cubic shaped lesukite. LDH, akaganeite, and the amorphous phases were of minor content. In the fractions > 2 µm the LDH compounds became besides lesukite also major phases. The amount of the other crystalline phases still remained less than 5 wt %. Residues of non-corroded fuel U_3_Si_2_, Fe (0), and lawrencite (Fe(II) compound) were exclusively present in the fraction >63 µm.

Depending on grain sizes fraction the content of amorphous phases varied and iron compounds with different valance states were observed. Although efforts were taken oxidation during the pre-treatment of the samples could not be ruled out and may hence explain the presence of Fe^3+^ in some compounds (cf. Table 1). This seemed notably true for the smaller grain sizes as within the treatment procedure this fraction was the more sensitive to alteration considering specific surface of the samples and the treatment duration. 

The amount of the amorphous phase could also be underestimated as during wet sieving some of the amorphous phases could be dissolved. This effect may even be increased for the fractions <63 µm because this material was additionally subdivided by the Atterberg method where further amorphous solids could be dissolved. 

In addition to corrosion rates future prospects of safety related issues of research reactor fuel elements must thus focus on the characterization of uranium with respect of its physicochemical properties in the amorphous part. This is an important issue considering the release and sorption of radionuclides within this uranium bearing solid. 

Furthermore, with respect to the corrosion rates the stability of each observed crystalline phase has to be determined individually. Consequently, it is important to get more insights into the physiochemical properties of lesukite in order to predict the sorption behavior of this compound for radionuclides under repository relevant conditions. First results with Eu^3+^ and SeO_4_^2−^ indicated a potential of retardation of anionic species (selenate) whereas europium interacts only weakly with lesukite [28]. The interaction of LDH phases with nuclear relevant compounds are also under investigation [41,42,43], because the incorporation of radioactive cations (e.g., cobalt, europium etc.) in the main layer and radioactive anions in the interlayer could lead to an immobilization these compounds. 

## Figures and Tables

**Table 1:** Phase quantities (crystalline, amorphous, and total) in dependence of the different grain size fractions and R_wp_ values of the Rietveld refinements. The left column also features the phase composition, phase name, and database (PDF-2 and ICSD) reference numbers of the identified crystalline phases.

Phase (PDF-2 No./ICSD No.)	Weight/%		
	<2 µm	2–63 µm	>63 µm
Al_2_Cl(OH)_5_·2H_2_O lesukite (00-031-0006/-)	95.37 ± 0.33	51.00 ± 2.75	13.87 ± 1.26
(Mg_0,67_Al_0,33_(OH)_2_)·(CO_3_)_0.165_·(H_2_O)_0.48_ LDH 3R (01-089-0460/86655)	1.43 ± 0.28	17.00 ± 4.08	7.37 ± 0.66
Al_2_Mg_4_(OH)_12_ (CO_3_) (H_2_O)_3_ LDH 2H 00-020-0658/82874		12.35 ± 1.33	10.21 ± 0.96
((Zn_0,625_ Al_0,375_) (OH)_2_) (SO_4_)_0.188_ LDH sulphate (01-070-6422/91859)	0.25 ± 0.08	3.94 ± 0.28	1.26 ± 0.19
(Fe(OH)_2_) ((OH)_0.25_ (H_2_O)_0.5_) green rust (00-040-0127/159700)		0.20 ± 0.06	2.15 ± 0.21
Fe_8_O_8_(OH)_8_Cl_1.35_ akaganeite (00-034-1266/69606)	2.05 ± 0.16	1.55 ± 0.15	0.65 ± 0.17
FeO(OH) goethite (01-081-0462/245057)		0.53 ± 0.08	
FeO(OH) lepidocrocite (01-070-8045/93948)		0.73 ± 0.11	
FeCl_2_ lawrencite (01-070-1634/64830)			0.68 ± 0.07
U_3_Si_2_ uranium silizide (00-005-0628/73695)			0.78 ± 0.07
Fe iron (00-006-0696/84483)			0.66 ± 0.10
Amorphous	0.90 ± 2.00	12.70 ± 5.30	62.36 ± 2.74
Total	100.00	100.00	100.00
Relative fraction amount (%)	20.30	8.20	71.50
R_wp_ (%)	12.21	8.20	0.95 *

* The very low weighted profile R-factor (R_wp_) (%) of 0.95% for the fraction >63 µm results from a manipulation of the background by adding stepwise intensity to the diffractograms in order to improve the description of the background by polynomials. The given R_wp_ is the mean value obtained by adding 100, 200, 300, 400, and 500 counts.
